# Coronaviruses in farm animals: Epidemiology and public health implications

**DOI:** 10.1002/vms3.359

**Published:** 2020-09-25

**Authors:** Médiha Khamassi Khbou, Monia Daaloul Jedidi, Faten Bouaicha Zaafouri, M’hammed Benzarti

**Affiliations:** ^1^ Laboratory of Infectious Animal Diseases, Zoonoses, and Sanitary Regulation Univ. Manouba. Ecole Nationale de Médecine Vétérinaire de Sidi Thabet Sidi Thabet Tunisia; ^2^ Laboratory of Microbiology and Immunology Univ. Manouba Ecole Nationale de Médecine Vétérinaire de Sidi Thabet Sidi Thabet Tunisia; ^3^ Department of Livestock Semiology and Medicine Univ. Manouba Ecole Nationale de Médecine Vétérinaire de Sidi Thabet Sidi Thabet Tunisia

**Keywords:** Coronaviruses, domestic animals, host jump, public health, widespread

## Abstract

Coronaviruses (CoVs) are documented in a wide range of animal species, including terrestrial and aquatic, domestic and wild. The geographic distribution of animal CoVs is worldwide and prevalences were reported in several countries across the five continents. The viruses are known to cause mainly gastrointestinal and respiratory diseases with different severity levels. In certain cases, CoV infections are responsible of huge economic losses associated or not to highly public health impact. Despite being enveloped, CoVs are relatively resistant pathogens in the environment. Coronaviruses are characterized by a high mutation and recombination rate, which makes host jumping and cross‐species transmission easy. In fact, increasing contact between different animal species fosters cross‐species transmission, while agriculture intensification, animal trade and herd management are key drivers at the human‐animal interface. If contacts with wild animals are still limited, humans have much more contact with farm animals, during breeding, transport, slaughter and food process, making CoVs a persistent threat to both humans and animals. A global network should be established for the surveillance and monitoring of animal CoVs.

## Graphical abstract

Animals coronaviruses cause mild to severe infections among farm animals, with high economic and public health impacts for some of them. Increasing demand in animal meat, animal trade and mixing different animal species together foster coronaviruses cross‐species transmission and expose human to new coronaviruses.

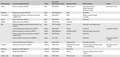

## 1 INTRODUCTION

The current pandemic of coronavirus disease (COVID‐19) raises several questions about the potential zoonotic role that domestic animals may play. Although the coronaviruses (CoVs) were described since several decades, today more than any time before, the fear of virus transmission from animals to humans is intensively discussed across the world. Until 27th August 2020, over 23 million persons were confirmed positive to the new severe acute respiratory syndrome coronavirus 2 (SARS‐CoV‐2) and nearly 800,000 lost their lives in an unpreceded pandemic, that stroke more than 210 countries and territories throughout the world (World Health Organization (WHO), 2020). While most of affected countries set up restrictive movements and policies inside and outside their boarders, and health systems are overwhelmed by severe caseload, the source of the virus remains elusive. The role of domestic animals in perpetuating the infection with CoVs to humans remains unclear. Several hypotheses have been advanced about the role of bat origin of the SARS‐CoV‐2, like it was the case of the SARS‐CoV and the Middle East respiratory syndrome (MERS‐CoV) (Salata, Calistri, Parolin, & Palù, 2020). The high genomic diversity among CoVs isolated from carnivores, herbivores and omnivores fosters the interspecies transmission and virus adaptation to new hosts (Song et al., 2005). In fact, cross‐species transmission and host jumping are the most important driver for virus emergence (Holmes, 2016) and CoVs specifically are dotted with a high rate of mutation and recombination, making them a continuous threat for humanity. Mutations result from errors during viral genome replication by the RNA‐dependent RNA polymerase, which reaches 5.7 × 10^–6^ nucleotide substitutions per site per day for the SARS‐CoV and the accumulated mutations at some sites could have important implication on virus properties (Vega et al., 2004). Recombination appears to be more important in CoV genome than mutation; it consists of gaining genome fragment from another CoV, which implies the adaptation to new hosts (Worobey & Holmes, 1999). As consequences of such genomic mutation and recombination the transmissible gastroenteritis virus (TGEV) of swine and the bovine CoV (BCoV) likely originated from the closely related canine coronavirus (CCoV) (Pratelli, 2011). A deletion that occurred in the S protein of the TGEV resulted in the rising of the porcine respiratory CoV with a marked changing tropism from the gastrointestinal to the respiratory tract (Peiris, 2012). There is evidence that the feline infectious peritonitis virus (FIPV) is originated from the feline enteric CoV (FECoV) by mutation in the region of the S1/S2 of the spike protein (Licitra et al., 2013).

The best opportunity that would allow viruses to jump new species occurs at the moment of feeding, when a predator consumes prey and all the viruses infecting it (French & Holmes, 2020). Beside feeding, physical contact between different host species increase host range expansion probability (Wang, Vlasova, Kenney, & Saif, 2019) as it was the case for the swine influenza viruses (subtypes H1N1 and H1N2) and the Nipah virus that were transmitted from pigs to humans (Chua et al., 2002; Gray et al., 2007). New porcine CoV emerged in China (Guangdong Province) in 2017, namely the swine acute diarrhoea syndrome CoV (SADS‐CoV) with high sequence identity to bat‐CoV HKU2 (Gong et al., 2017). The high density of pig farms and slaughterhouses in Guangdong Province associated to the wide distribution of bat species explained the cross‐species transmission. Indeed, as pork meat is considered as the most commonly consumed meat in non‐Muslim countries, pigs may be an effective intermediate host for the emergence of novel CoVs of highly public health concerns (Wang, Su, Bi, Wong, & Gao, 2018).

Coronaviruses of farm animals including large and small ruminants, dromedaries, horses, pigs and chickens were reviewed; cetacean CoVs were also considered, as marine mammals are a food source in many countries around the world. The aim of this review is to summarize the epidemiological knowledge about CoVs of farm animals, to discuss the public health implications and to address the gap of knowledge useful for future research direction.

## 2 VIROLOGY

**FIGURE 1:**
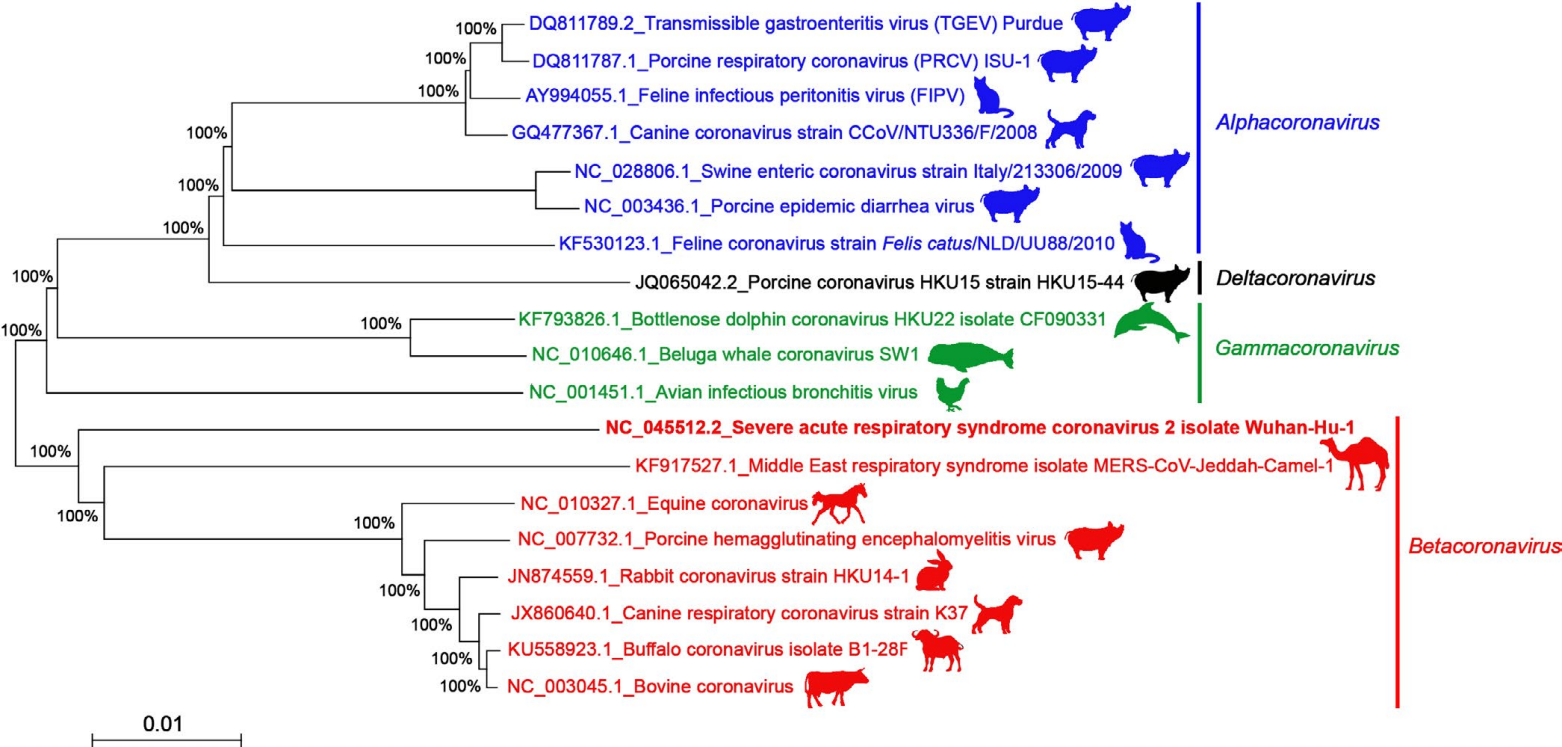
Phylogenetic tree of different representative CoVs based on the Spike (S) protein gene (the strain responsible for the pandemic SARS‐CoV‐2 in humans is shown with the bolded character among the *Betacoronavirus* genus). The shown animals are hosts from where the corresponding CoVs were isolated. The sequences of all animal CoVs were downloaded from the National Center for Biotechnology Information (NCBI) GenBank database (https://www.ncbi.nlm.nih.gov/genbank/). The tree was constructed using the Neighbour‐Joining method (bootstrap resampling = 1,000 replicates and bootstrap values are indicated as % at branch points) (Saitou & Nei, 1987). The evolutionary distances were computed using the Maximum Composite Likelihood method (Tamura et al., 2004) and are in the units of the number of base substitutions per site. All positions containing either gaps or missing data were eliminated. There were a total of 2044 positions in the final dataset. Evolutionary analyses were made with MEGA7 (Kumar et al., 2016)

Coronaviruses (CoVs) belong to the subfamily of Coronavirinae within the family Coronaviridae and the order Nidovirales (MacLachlan & Dubovi, 2017). Coronaviruses are enveloped viruses with a single stranded, non‐segmented and positive RNA, of 27 to 31 kb, they are the biggest among all RNA viruses (Lai et al., 1994). The family of Coronaviridae consists of four genera: *Alphacoronavirus* (Alpha‐CoV), *Betacoronavirus* (Beta‐CoV), *Gammacoronavirus* (Gamma‐CoV) and *Deltacoronavirus* (Delta‐CoV) (Fehr & Perlman, 2015). Most of the mammalian CoVs belong to Alpha‐ and Beta‐CoV (Su et al., 2016), whereas the avian and the cetacean CoVs are in the Gamma‐CoV (Table 1, Figure 1).

**TABLE 1:** List of the main coronaviruses infecting farm animals

Animal species	Coronavirus (abbreviation)	Genus	Year of first description (country)	Disease severity	Main symptoms	Vaccine
Cattle	Bovine coronavirus (BCoV)	Beta	1973 (USA)	Mild to severe	Neonatal diarrhoea Winter dysentery Respiratory signs	Inactivated or MLV
Buffaloes	Bubaline coronavirus (BuCoV)	Beta	1985 (Bulgaria)	Mild	Diarrhoea	
Dromedaries	Middle East respiratory syndrome coronavirus (MERS‐CoV)	Beta	2012 (KSA)	Mild	Rhinitis, nasal discharge Tracheitis	
Horse	Equine coronavirus (ECoV)	Beta	1999 (USA)	Mild	Diarrhoea	
Rabbits	Rabbit coronavirus (RbCoV)	Beta	2012 (China)	Subclinial		
Pigs	Porcine hemagglutinating encephalomyelitis virus (PHEV)	Beta	1957 (Canada)	Severe	Diarrhoea, neurological signs	
	Transmissible gastroenteritis virus (TGEV)^a^	Alpha	1946 (USA)	Severe	Diarrhoea	Inactivated or MLV
	Porcine respiratory coronavirus (PRCV)	Alpha	1983 (Belgium)	Subclinical‐Mild	Nasal discharge, pneumonia	
	Swine acute diarrhoea syndrome coronavirus (SADS‐CoV)	Alpha	2016 (China)	Severe	Diarrhoea	
	Porcine epidemic diarrhoea virus (PEDV)	Alpha	1971 (UK)	Severe	Diarrhoea	Inactivated or MLV or VBV
	Porcine delatcoronavirus (PDCoV)	Delta	2009 (China)	Mild to severe	Diarrhoea, vomiting	
Chickens	Infectious bronchitis virus (IBV)^a^	Gamma	1930 (USA)	Mild (adults) to severe (young)	Nasal discharge, snicking, watery eyes and lethargy	Inactivated or MLV or VBV
Dolphine	Bottlenoise whale (BdCoV)	Gamma	2014 (China)	Subclinical		
Whale	Beluga whale (BWCoV)	Gamma	2008 (USA)	Severe	Pulmonary disease and acute liver failure	
Harbor seals	Not assigned yet	Alpha	1987 (USA)	Mild to severe	Acute enteritis	

Abbreviations: Alpha, *Alphacoronavirus*; Beta, *Betacoronavirus*; Delta, *Deltacoronavirus*; Gamma, *Gammacoronavirus*; KSA, Kingdom of Saudi Arabia; MLV, modified live vaccine; UK, United Kingdom; USA, United States of America; VBV, vector based vaccine.

^a^ Notifiable disease to the World Organisation of Animal Health (OIE).

A full description of CoVs structure, replication, phylogenetic, evolution and pathogenesis are available in previous comprehensive reviews (Li, 2016; Schoeman & Fielding, 2019; Weiss & Leibowitz, 2011; Yang & Leibowitz, 2015). The main characteristics of CoVs are summarized in the BOX 1.

BOX 1 **Properties of coronaviruses**

**Table:** 

*Morphology*: spherical (80–220 nm) *Envelop*: bilayer lipid *Genome type*: single stranded positive RNA, infectious *Genome size*: 27–31 kb *Replication site*: cytoplasm *Recombination rate*: very high (10^–4^ substitution per year and per site) *Structural proteins*: envelope (E), membrane (M), spike (S), nucleocapsid (*N*) and in few coronaviruses hemagglutinin‐esterase (HE) *Main antigenic protein*: spike protein *Main immunogenic protein*: spike protein *Most conserved genes*: the envelope (E) and membrane (M) protein genes *Most variable genes*: the spike (S) protein genes *Resistance*: 9 days in dry state for the SARS‐CoV, 22 days in water at 25°C for the TGEV, 7 days for the MHV (mouse hepatitis virus) in sewage *Susceptibility*: heat (30 min at 56°C), alcohol based solutions (30 s to 1 min)

## 3 EPIDEMIOLOGY OF FARM ANIMALS’ CORONAVIRUSES

### 3.1 Coronaviruses of Bovidae

#### 3.1.1 Cattle

Bovine coronavirus (BCoV) was first associated with neonatal calves diarrhoea by Mebus et al. in the United States (1973). To date, 38 BCoV genomes were published in GenBank (He et al., 2019). The BCoV is the causative agent of winter dysentery (WD) in adult cattle (Saif, Redman, Brock, Kohler, & Heckert, 1988) and causes respiratory tract infections in calves, cattle and small ruminants (Storz et al., 2000). Recent reports identified differences in antigenic, genomic and culture characteristics between respiratory and enteric BCoV strains (Vilček, Jacková, Kolesárová, & Vlasáková, 2017). Several Bovine‐like coronaviruses were detected in domestic animals and were reviewed by Amer (2019).

**FIGURE 2:**
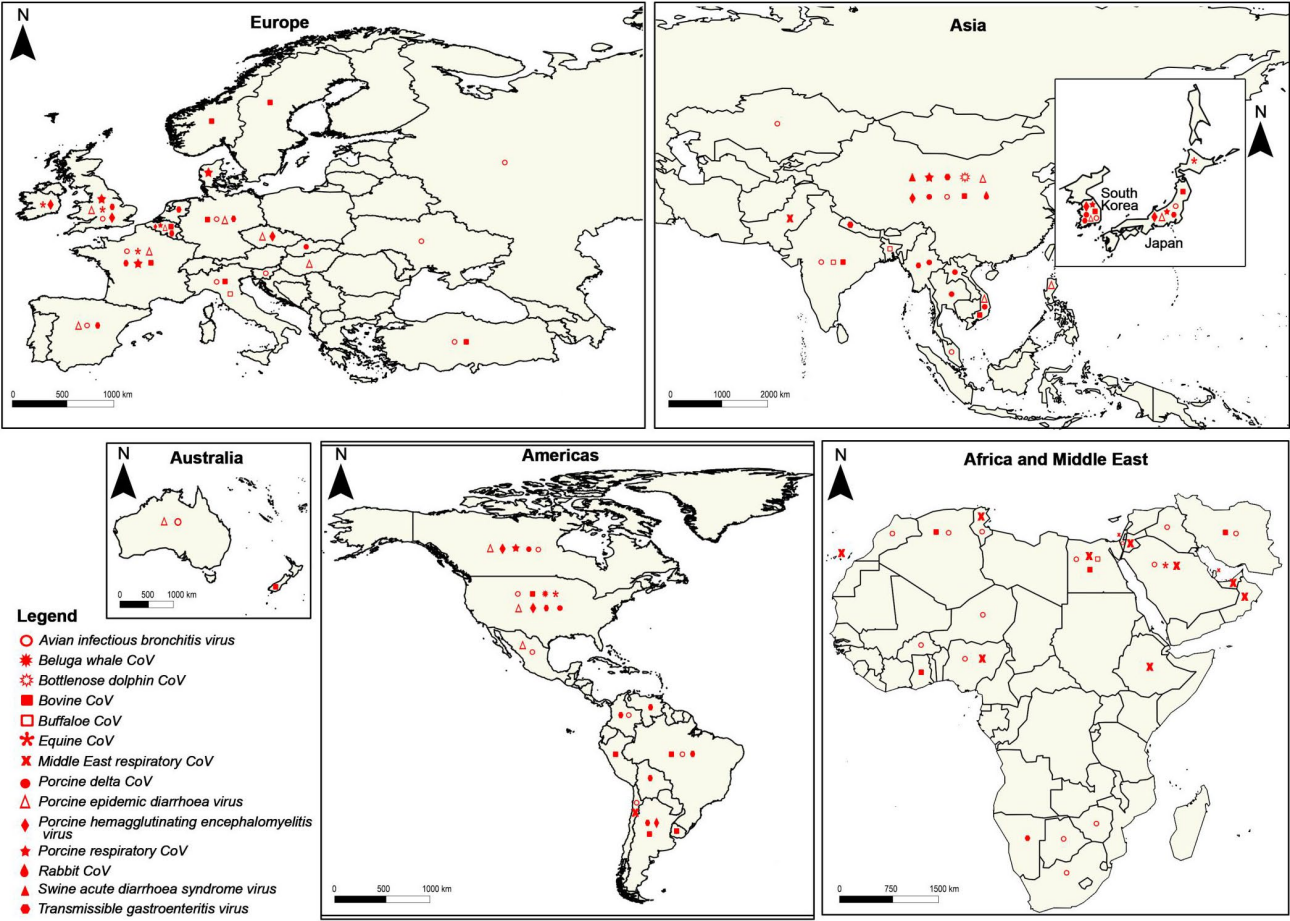
The map of the five continents, showing the countries where different animal coronaviruses were detected either using serological or molecular tools. The map was created in QGIS 3.12.2 (QGIS, 2018)

The seroprevalence of anti‐BCoV antibodies among newborn diarrheic calves ranged from 20% (17/82) in Algeria (Ammar et al., 2014) to 93.9% (172/183) in Turkey (Yavru et al., 2018). Molecular detection of the virus by qRT‐PCR was reported from several countries leading to variable prevalences (Figure 2, Table 2). In Vietnam, 16 out of 232 (6.9%) diarrheic calves were tested positive and the genetic characterization of S and HE proteins reveals that Vietnam BCoV sequences might share a common ancestor with Cuban and Chinese BCoVs (Shin et al., 2019). Recently, the occurrence of BCoV in neonatal calf diarrhoea was estimated to 7.2% (14/194) in Iran using antigen‐capture ELISA (Lotfollahzadeh et al., 2020).

**TABLE 2:** A collective summary of coronaviruses in farm animals

*Animal family* Animal species (clinical status)	Number of positive samples/Total samples tested (%)	Country (State/Province)	Type of sample	Detection method	Targeted gene for molecular tests or used antigen for serological tests	Coronavirus	Authors
*Bovidae*							
Cattle (respiratory disease)	36/46 (78.2)	Italy (Calabria, Apulia)	Nasal	RT‐PCR/qRT‐PCR	*S* gene/*M* gene	BCoV	Decaro, Campolo, et al. (2008)
Cattle (respiratory disease)	35/36 (97.2)	Italy (Calabria, Apulia)	Rectal	RT‐PCR/qRT‐PCR	*S* gene/*M* gene	BCoV	Decaro, Campolo, et al. (2008)
Cattle (respiratory disease)	11/202 (5.4)	Belgium (Flanders)	Nasal	ELISA	*N*.*M*.	BCoV	Pardon et al.(2011)
Cattle and water buffalo (diarrheic)	15/160 (9.38)	India (Uttar Pradesh, Tamil Nadu, Karnataka, Bareilly)	Faecal	RT‐PCR	*N* gene	BCoV	Santhosh Kumar et al. (2012)
Cattle (diarrheic)	17/82 (20.7)	Algeria (Tiaret)	Faecal	ELISA indirect Ag‐capture	*S* protein	BCoV	Ammar et al. (2014)
Cattle^b^	102/602 (17)	France	Respiratory	qRT‐PCR	*N*.*M*.	BCoV	Meyer et al. (2015)
Cattle^b^ (respiratory disease)	?/? (63.2)	France	Nasal	qRT‐PCR	*N*.*M*.	BCoV	Meyer et al. (2015)
Cattle^b^ (diarrheic)	92/5,365 (1.7)	Argentina	Faecal	ELISA indirect Ag‐capture	*HE*, *N*, S proteins	BCoV	Bok et al. (2015)
Cattle	72/1,226 (5.8)	New Zealand (7 regions)	Rectal	ELISA	*N*.*M*.	BCoV	Al Mawly et al. (2015)
Cattle	973/1,347 (72.2)^c^	Norway (Sogn og Fjordane, Møre og Romsdal)	Milk	ELISA	*N*.*M*.	BCoV	Toftaker, Sanchez, Stokstad, and Nødtvedt (2016)
Cattle (diarrheic)	3/25 (12)	Thailand (Central)	Blood + Faeces	RT‐PCR + Partial sequencing	*S* gene		Singasa et al. (2017)
Cattle	47/101 (46.5)	Brazil (Rio de Janeiro)	Faecal	Pan‐RT‐PCR + Partial sequencing	Polymerase gene	BCoV	Rocha et al. (2018)
Cattle (diarrheic)	1/184 (0.5)	Turkey (Burdur)	Faecal	ELISA direct	*N*.*M*.	BCoV	Yavru et al. (2018)
Cattle (diarrheic)	172/184 (93.5)	Turkey (Burdur)	Sera	ELISA indirect	*N*.*M*.	BCoV	Yavru et al. (2018)
Cattle (diarrheic)	16/232 (6.9)	Vietnam (9 provinces)	Faecal	RT‐PCR + Complete sequencing	*N* gene	BCoV	Shin et al. (2019)
Cattle (diarrheic)	15/37 (40.5)	Egypt (Ismailia, Al‐Sharqya, Alexandria)		qRT‐PCR	*M* gene	BCoV	El‐Sadek et al. (2019)
Cattle (diarrheic and healthy)	64/824^b^ (7.8)	Uruguay (11 districts)	Faecal	RT‐PCR + Partial sequencing	*S* gene	BCoV	Castells et al. (2019)
Cattle (diarrheic)	13/207 (6.2)	South Korea (Gangwon, Gyeongbuk)	Faecal	qRT‐PCR	*N*.*M*.	CoV	Lee et al. (2019)
Cattle (diarrheic)	36/190 (18.9)	China (6 provinces)	Faecal	RT‐PCR + Partial sequencing	Polymerase gene	BCoV	Keha et al. (2019)
Cattle (diarrheic and healthy)	37/689 (5.4)	South Korea (9 regions)	Faecal	RT‐PCR + Partial sequencing	*S* gene	BCoV	Ryu et al. (2020)
Cattle (apparently healthy)	823/1,495 (55)	Ghana (5 districts)	Sera	IFA	*S* protein	BCoV	Burimuah et al. (2020)
Cattle (diarrheic)	14/194 (7.2)	Iran (10 provinces)	Faecal	Antigen capture ELISA	*N* and *S* genes	BCoV	Lotfollahzadeh et al. (2020)
Water buffaloe	10/?	Italy (Caserta, Latina, Foggia)	Faecal + Intestinal	RT‐PCR + Partial sequencing	S gene	BuCov	Decaro et al. (2010b)
Water buffaloe	2/?	Bangladesh	Faecal	RT‐PCR + Complete sequencing	*RdRp* gene	BufCoV HKU26	Lau et al. (2016)
Water buffaloe (diarrheic)	7/45 (15.6)	Egypt (Ismailia, Al‐Sharqya, Alexandria)	Faecal	qRT‐PCR	*M* gene	BCoV	El‐Sadek et al. (2019)
Yak (diarrheic)	232/336 (69.05)	China (Tibet, Qinghai, Sichuan, Yunnan)	Faecal	RT‐PCR + Complete sequencing	*S* + *HE*+*N* genes	BCoV	He et al. (2019)
Sheep (apparently healthy)	42/218 (19.2)	Sweden	Sera	ELISA	*N*.*M*.	BCoV	Tråvén et al. (1999)
Sheep (apparently healthy)	26/102 (25.5)	Ghana (5 districts)	Sera	IFA IgG	*S* protein	BCoV	Burimuah et al. (2020)
Goats (apparently healthy)	29/66 (43.1)	Ghana (5 districts)	Sera	IFA IgG	*S* protein	BCoV	Burimuah et al. (2020)
*Camelidae*							
Camels (apparently healthy)	50/50 (100)	Oman	Sera	Protein microarray	*S1* protein	MERS‐CoV	Reusken et al. (2013)
Camels (apparently healthy)	15/105 (14.3)	Spain (Canary Island)	Sera	Protein microarray	*S1* protein	MERS‐CoV	Reusken et al. (2013)
Camels (apparently healthy)	337/358 (94)	Nigeria (Kano, Sokoto, Borno, Adamawa)	Sera	Protein microarray	*S1* protein	MERS‐CoV	Reusken, Messadi, et al. (2014)
Camels (apparently healthy)	182/188 (97)	Ethiopia (Afar, Somalia, Oromia)	Sera	Protein microarray	*S1* protein	MERS‐CoV	Reusken, Messadi, et al. (2014)
Camels (apparently healthy)	89/204 (43.6)	Tunisia (Sousse, Sidi Bouzid, Kébili)	Sera	Protein microarray	*S1* protein	MERS‐CoV	Reusken, Messadi, et al. (2014)
Camels (apparently healthy)	1/53 (1.9)	Qatar (Doha)	Nasal	RT‐PCR + Complete sequencing	*E* and *N* genes	MERS‐CoV	Raj et al. (2014)
Camels (apparently healthy)	126/7,803 (1.6)	UAE (Abu Dhabi)	Nasal	qRT‐PCR + Partial sequencing	*E* and *ORF1* genes	MERS‐CoV	Yusof et al. (2015)
Camels (apparently healthy)	1,808/2,541 (71)	Egypt (5 districts)	Sera	Microneutralization assay	*S* protein	MERS‐CoV	Ali et al. (2017)
Camels (apparently healthy)	435/2,825 (15.4)	Egypt (5 districts)	Nasal	qRT‐PCR + Partial sequencing	*E* gene	MERS‐CoV	Ali et al. (2017)
Camels (apparently healthy)	18/114 (15.8)	Egypt (5 districts)	Rectal	qRT‐PCR + Partial sequencing	*E* gene	MERS‐CoV	Ali et al., (2017)
Camels (apparently healthy)	12/187 (6.4)	Egypt (5 districts)	Milk	qRT‐PCR + Partial sequencing	*E* gene	MERS‐CoV	Ali et al. (2017)
Camels (apparently healthy)	37/45 (82)	Jordan (Zarqa, Ramtha)	Sera	ELISA	*S* protein	MERS‐CoV	Van Doremalen et al. (2017)
Camels (apparently healthy)	42/45 (93.3)	Jordan (Zarqa, Ramtha)	Nasal	qRT‐PCR + Partial sequencing	*E* + *ORF1* genes	MERS‐CoV	Van Doremalen et al. (2017)
Camels (apparently healthy)	792/1,163 (68)	Kenya (country wide)	Plasma	Indirect ELISA (IgG)	*S* protein	MERS‐CoV	Ommeh et al. (2018)
Camels (apparently healthy)	11/1,163 (0.9)	Kenya (country wide)	Nasal	qRT‐PCR + Complete sequencing	*N* gene	MERS‐CoV	Ommeh et al. (2018)
Camels (apparently healthy)	794/1,050 (75.6)	Pakistan (Punjab, Sindh, Balochistan, Khyber)	Sera	Indirect ELISA (IgG)	*S* protein	MERS‐CoV	Zohaib et al. (2018)
Camels (apparently healthy)	22/776 (2.8)	Pakistan (Punjab, Sindh, Balochistan, Khyber)	Nasal	qRT‐PCR + Nested RT‐PCR	*S* + *N* genes	MERS‐CoV	Zohaib et al. (2018)
Camels (apparently healthy)	144/171 (84)	KSA (Tabuk)	Sera	ELISA (IgG)	*S1* protein	MERS‐CoV	Harrath and Abu Duhier (2018)
Camels (apparently healthy)	31/38 (81.5)	Ethiopia (Bati, Fafen)	Sera	Neutralization test	*N*.*A*.	MERS‐CoV	Teramichi et al. (2019)
Llamas and alpacas (diarrheic)	19/45 (42)	USA (Oregon)	Faecal	Electron microscopy	*N*.*A*.	CoVs	Cebra et al. (2003)
Llamas and alpacas	0/25 (0)	Chile	Sera	Protein microarray	*S1* protein	MERS‐CoV	Reusken et al. (2016)
Alpacas (diarrheic)	20/50^b^ (40)	Peru (Silli)	Intestinal	RT‐PCR	Polymerase gene	CoVs	Rojas et al. (2016)
Alpacas (healthy)	15/15 (100)	Qatar (Al‐Shahanyia)	Sera	PRNT50	*N*.*A*.	MERS‐CoV	Reusken et al. (2016)
Alpacas (healthy)	70/261 (26.8)	Peru (Cuzco)	Faecal	Pan‐RT‐PCR + Partial sequencing	Polymerase gene	BCoV	Rocha et al. (2018)
*Equidae*							
Horses (enteric disease)	5/10 (50)	Japan (Hokkaido)	Faecal	RT‐PCR + Partial sequencing	*N* gene	ECoV	Oue et al. (2013)
Horses (sick)	38/44 (86)	USA (4 States)	Faecal	qRT‐PCR + Partial sequencing	*N* gene	ECoV	Pusterla et al. (2013)
Horses (apparently healthy)	7/96 (7.3)	USA (4 States)	Faecal	qRT‐PCR + Partial sequencing	*N* gene	ECoV	Pusterla et al. (2013)
Horses (apparently healthy)	10/37 (27)	USA (Kentucky)	Faecal	qRT‐PCR	*M* gene	ECoV	Slovis, Elam, Estrada, and Leutenegger (2014)
Horses (gastrointestinal disease)	15/51 (29.4)	USA (Kentucky)	Faecal	qRT‐PCR	*M* gene	ECoV	Slovis et al. (2014)
Horses (enteric mild disease)	11/395 (2.8)	France (58 counties)	Faecal	qRT‐PCR + Partial sequencing	*M* and *N* genes	ECoV	Miszczak et al. (2014)
Horses (respiratory mild disease)	1/200 (0.5)	France (58 counties)	Respiratory	qRT‐PCR + Partial sequencing	*M* and *N* genes	ECoV	Miszczak et al. (2014)
Horses^a^(respiratory disease)	17/2,437 (0.7)	USA	Nasal	qRT‐PCR	*N* gene	ECoV	Pusterla et al. (2015)
Horses^a^ (healthy)	0/187 (0)	USA	Nasal	qRT‐PCR	*N* gene	ECoV	Pusterla et al. (2015)
Horses (diarrheic)	0/307 (0)	Japan (Hokkaido)	Rectal	RT‐LMIA + qRT‐PCR + Partial sequencing	*N* gene	ECoV	Nemoto et al. (2015)
Horses (healthy)	3/120 (2.5)	Japan (Hokkaido)	Rectal	RT‐LMIA + qRT‐PCR + Partial sequencing	*N* gene	ECoV	Nemoto et al. (2015)
Miniature horse	15/27 (55.5)	USA (California, Idaho)	Faecal	qRT‐PCR	*N* gene	ECoV	Fielding et al. (2015)
Horses (apparently healthy)	4/297	KSA (6 cities)	Rectal	qRT‐PCR + Partial sequencing	*M* and *N* genes	ECoV	Hemida, Chu, et al. (2017)
Horses (apparently healthy)	0/298	KSA (6 cities)	Nasal	qRT‐PCR + Partial sequencing	*M* and *N* genes	ECoV	Hemida, Chu, et al. (2017)
Horses (apparently healthy)	504/5,247 (9.6)	USA (18 States)	Sera	IgG ELISA	*S* protein	ECoV	Kooijman et al. (2017)
Horses (with symptoms)	4/94 (4.2)	UK	Faecal	qRT‐PCR	*N* gene	ECoV	Bryan et al. (2019)
Horses (apparently healthy)	0/225 (0)	UK	Faecal	qRT‐PCR	*N* gene	ECoV	Bryan et al. (2019)
Donkeys	0/62 (0)	UK	Faecal	qRT‐PCR	*N* gene	ECoV	Bryan et al. (2019)
Horses (gastrointestinal disease)	1/65 (1.5)	USA (Washington)	Faecal	qRT‐PCR	*N* gene	ECoV	Sanz et al. (2019)
Horses (apparently healthy)	0/65 (0)	USA (Washington)	Faecal	qRT‐PCR	*N* gene	ECoV	Sanz et al. (2019)
Miniature horse	25/29 (86)	USA (New York)	Faecal	RT‐PCR	*N* gene	BetaCoV	Goodrich et al. (2020)
*Suidae*							
Swine	316/347 (91)	USA (Iowa)	Sera	Serum neutralization test	*N*.*M*.	TGEV/PRCV	Wesley, Woods, McKean, Senn, and Elazhary (1997)
Swine (enteric and neurological signs)	16/16 (100)	Argentina	Brain tissu	RT‐PCR	Polymerase gene	PHEV	Quiroga et al. (2008)
Swine (healthy)	237/446 (53.1)	South Korea (5 States)	Sera	Blocking ELISA	*N*.*M*.	PRCV	Chae et al. (2000)
Swine (with enteric or respiratory or neurological signs)	22/239 (9.2)	South Korea	Faecal	Nested RT‐PCR + Partial sequencing	*N* gene	PHEV	Rho et al. (2011)
Swine (no signs)	585/1,117 (52.4)	China (Jilin)	Sera	Immunochromatographic strip	*HE* protein	PHEV	Chen et al. (2011)
Swine	17/169 (10)	China (Hong Kong)	Rectal	RT‐PCR + Complete sequencing	*RdRp* gene	PDCoV HKU15	Woo et al. (2012)
Swine (diarrheic)	19/109 (17.4)	USA (9 States)	Faecal	qRT‐PCR	*M* gene	PEDV	Wang et al. (2014b)
Swine (diarrheic)	109/435 (25)	USA (9 States)	Faecal	qRT‐PCR + Complete sequencing	*M* gene	PDCoV	Wang et al. (2014b)
Swine (diarrheic)	39/42 (93)	USA (Ohio)	Faecal + Intestinal	RT‐PCR + Complete sequencing	*M* gene	PDCoV	Wang et al. (2014a)
Swine (diarrheic)	5/42 (11.9)	USA (Ohio)	Faecal + Intestinal	qRT‐PCR	*M* gene	PEDV	Wang et al. (2014a)
Swine^a^	2/100 (2)	South Korea (Gyeonbuk)	Faecal + Intestinal	RT‐PCR + partial sequencing	*N* gene		Lee et al. (2016)
Swine (acute diarrhoea)	26/30 (86.6)	Thailand (East)	Blood, lymph nodes, faeces, feed	RT‐PCR + Complete sequencing	*M* et *N* genes	PDCoV	Janetanakit et al. (2016)
Swine (history of diarrhoea)	55/252 (21.8)	China (Guangdong)	Faecal + Intestinal	RT‐PCR + Partial sequencing	*N* and *S* genes	PDCoV HKU16	Mai et al. (2018)
Swine (history of diarrhoea)	165/252 (65.5)	China (Guangdong)	Faecal + Intestinal	RT‐PCR	*E* gene	PEDV	Mai et al. (2018)
Swine (history of diarrhoea)	0/252 (0)	China (Guangdong)	Faecal + Intestinal	RT‐PCR	*S* gene	TGEV	Mai et al. (2018)
Swine (diarrhoea)	10/34 (29.4)	Philippines (Luzon Island)	Faecal	RT‐PCR + Partial sequencing	*N* gene	PEDV	Garcia et al. (2018)
Swine (no history of diarrhoea)	12/151 (7.9)	Czech Republic	Nasal	RT‐PCR + Complete sequencing	*HE* and *S* genes	PHEV	Moutelikova and Prodelalova (2019)
Swine (diarrheic)	5/215 (2.3)	Spain (Catalonia)	Faecal	qRT‐PCR	*N* gene	PEDV	Vidal et al. (2019)
Swine (diarrheic)	6/215 (2.8)	Spain (Catalonia)	Faecal	qRT‐PCR	*N* gene	TGEV	Vidal et al. (2019)
Swine^a^ (history of diarrheic disease)	74/170 (43.5)	China (Guangdong)	Archived (Faecal + Intestinal)	RT‐PCR	*N* gene	SADS‐CoV	Zhou, Li, et al. (2019)
Swine^a^ (history of diarrheic disease)	133/170 (78.2)	China (Guangdong)	Archived (Faecal + Intestinal)	RT‐PCR	*N* and *S* genes	PEDV	Zhou, Li, et al. (2019)
Swine^a^ (history of diarrheic disease)	53/236 (22.5)	China (Guangdong)	Faecal	RT‐PCR + Partial sequencing	*N* gene	SADS‐CoV	Zhou, Li, et al. (2019)
Swine^a^ (history of diarrheic disease)	15/170 (8.8)	China (Guangdong)	Archived (Faecal + Intestinal)	RT‐PCR	*N* and *S* genes	PDCoV	Zhou, Li, et al. (2019)
Swine (healthy)	13/18 (72.2)	China (Guangdong)	Intestinal	ELISA	*N*.*M*.	SADS‐CoV	Zhou, Sun, et al. (2019)
Swine^a^ (diarrheic)	1,712/2,987 (57.3)	China (5 provinces)	Faecal + Intestinal +Milk	RT‐PCR + Partial sequencing	*ORF1* gene	PEDV	Zhang, Luo, et al. (2019)
Swine^a^ (diarrheic)	813/2,987 (27.2)	China (5 provinces)	Faecal + Intestinal +Milk	Nested RT‐PCR	*N* gene	PDCoV	Zhang, Luo, et al. (2019)
Swine^a^ (diarrheic)	21/2,987 (0.7)	China (5 provinces)	Faecal + Intestinal +Milk	RT‐PCR	*S* gene	TGEV	Zhang, Luo, et al. (2019)
Swine^a^ (diarrheic)	7/2,987 (0.2)	China (5 provinces)	Faecal + Intestinal +Milk	RT‐PCR	*N* gene	PEDV	Zhang, Luo, et al. (2019)
Swine (diarrheic and healthy)	74/193 (38.3)	China (Qinghai‐Tibetan Plateau)	Faecal	RT‐PCR + Complete sequencing	*N* gene	PEDV	Qin et al. (2019)
Swine (diarrheic and healthy)	0/193 (0)	China (Qinghai‐Tibetan Plateau)	Faecal	RT‐PCR	*S* gene	TGEV/PDCoV	Qin et al. (2019)
Swine^a^ (history of diarrhoea)	11/108 (10.1)	Vietnam	Intestinal	RT‐PCR + Complete sequencing	*M* and *N* genes	PDCoV	Saeng‐Chuto et al. (2020)
Swine^a^ (history of diarrhoea)	87/108 (80.5)	Vietnam	Intestinal	RT‐PCR	*S* gene	PEDV	Saeng‐Chuto et al. (2020)
Swine^a^	0/398 (0)	China (9 provinces)	Faecal	Multiplex RT‐PCR	*N* gene	TGEV	Ding et al. (2020)
Swine^a^	97/398 (24.3)	China (9 provinces)	Faecal	Multiplex RT‐PCR	*N* gene	PEDV	Ding et al. (2020)
Swine^a^	157/398 (39.4)	China (9 provinces)	Faecal	Multiplex RT‐PCR	*N* gene	PDCoV	Ding et al. (2020)
Swine^a^ (history of diarrhoea)	0/108 (0)	Vietnam	Intestinal	RT‐PCR	*N* gene	TGEV	Saeng‐Chuto et al. (2020)
Swine^a^ (no history of disease)	1,470/2,756 (53.35)	USA (19 States)	Sera	ELISA	*S1* protein	PHEV	Mora‐Díaz et al. (2020)
Swine (diarrheic and healthy)	53/68 (78)	Mexico (15 States)	Faecal	qRT‐PCR + Partial sequencing	*S* gene	PEDV	Reveles‐Félix et al. (2020)
Swine (diarrheic and healthy)	70/184 (38)	China (9 provinces)	Faecal	RT‐PCR + Partial sequencing	*S* gene	PEDV	Tan et al. (2020)
Swine (diarrheic and healthy)	6/184 (3.2)	China (9 provinces)	Faecal	RT‐PCR	*S* gene	TGEV	Tan et al. (2020)
Swine^a^	12/58 (20.7)	China (Shandong)	Faecal + Intestinal	RT‐PCR + Partial sequencing	S, *N*, *ORF1* genes	PDCoV	Sun et al. (2020)
Swine^a^	20/58 (34.5)	China (Shandong)	Faecal + Intestinal	RT‐PCR	*N*.*M*.	PEDV	Sun et al. (2020)
*Leporidae*							
Rabbit (healthy)	11/136 (8.1)	China (Guangzhou)	Faecal	qRT‐PCR + Complete sequencing	*RdRp* gene	RbCoV HKU14	Lau et al. (2012)

Abbreviations: BCoV, Bovine coronavirus; BuCoV, Bubaline coronavirus; BufCoV, Buffaloe coronavirus; CoV, coronavirus; E, Envelope; ECoV, Equine coronavirus; ELISA, Enzyme‐linked immunosorbent assay; HE, Hemagglutinin esterase; IFA, Immunofluorescence assay; KSA, Kingdom of Saudi Arabia; LMIA, Loop mediated isothermal amplification; M, Membrane; MERS‐CoV, Middle East respiratory syndrome coronavirus; *N*, Nucleocapside; *N*.A, not applicable; *N*.M., not mentioned; ORF, Open reading frame; PDCoV, Porcine deltacoronavirus; PEDV, Porcine epidemic diarrhoea virus; PHEV, Porcine hemagglutinating encephalomyelitis virus; PRCV, Porcine respiratory coronavirus; qRT‐PCR, quantitative RT‐PCR; RbCoV, Rabbit coronavirus; RdRp, RNA dependant RNA polymerase; RT‐PCR, Reverse transcriptase polymerase chain reaction; S, Spike; TGEV, Transmissible gastroenteritis virus; UAE, United Arab Emirates; UK, United Kingdom; USA, United States of America.

?: Unknown numerator or denominator;

^a^ Retrospective study

^b^ Samples comprised those from dead animals

^c^ Herd prevalence.

Calves and cattle with either diarrhoea or respiratory infections are the main source of the virus (Saif, 2010) and infection by BCoV occurs either by faecal‐oral or aerosol contamination. The BCoV is shed both through respiratory and enteric secretions in high amounts (1 billion virus particles per ml of faeces) for up to 14 days (Kapil et al., 1990). Some studies showed that the virus persists in sub‐clinically infected adult cattle (Park et al., 2007; Toftaker, Holmøy, Nødtvedt, Østerås, & Stokstad, 2017). Furthermore, high animal density seems to be the main BCoV risk factors (Boileau & Kapil, 2010).

The BCoV is one of the main causative agents of neonatal calf diarrhoea during the first month of life (Ammar et al., 2014; Brandão et al., 2006) and the most common deathly disease in calves (Gomez & Weese, 2017). Other studies showed that BCoV may be involved in 10 to 30% of neonatal diarrhoea cases (Alfieri et al., 2018).

In adult cattle, the WD occurs as epidemics during winter and is characterized by a contagious watery diarrhoea, fever, depression and reduced milk yield (Toftaker et al., 2017). Singasa, Songserm, Lertwatcharasarakul, and Arunvipas (2017) reported that milk production might decrease up to 10% for 2 weeks during the BCoV infection.

Vaccines are available for the prevention of BCoV infections in neonatal calves and in adult. An inactivated virus vaccine was developed for pregnant cows to enhance passive maternal immunization of calves via colostrum and prevent neonatal diarrhoea (Decaro et al., 2009). Another inactivated hemagglutinating antigens‐enriched vaccine showed their efficacy to protect cattle against WD (Takamura, Matsumoto, & Shimizu, 2002).

The BCoV was also associated, alone or with other pathogens, to respiratory infections such as enzootic pneumonia in calves and shipping fever in young cattle (Saif, 2010). The prevention from respiratory CoV in cattle is based on multivalent killed or attenuated‐live intra‐nasally administrated vaccines (Hay et al., 2016; Richeson et al., 2019).

#### 3.1.2 Water buffaloes

A bovine‐like CoV in water buffalo (*Bubalus bubalis*) was first described in 1985 in Bulgaria, using serological methods (Muniiappa, Mitov, & Kharalambiev, 1985). The sequencing of the isolated buffalo CoV from Italy, showed sequence homology with the BCoV, with some differences justifying its classification as a new variant of BCoV and was named as Bubaline CoV (BuCoV) (Decaro, Martella, et al., 2008). In Italy, gastroenteritis disease among water buffalo calves is caused by both BuCoV and BCoV (Decaro et al., 2010). Closely related strains to the BCoV (displaying 98% sequence homology) were also detected among faecal samples of domestic buffaloes in Bangladesh (Lau et al., 2016). Infection of water buffalo calves was also reported from three districts in Egypt, with an overall molecular prevalence of 26.8% (22/82) in faecal samples (El‐Sadek et al., 2019) (Table 2).

#### 3.1.3 Small ruminants

Although the BCoV is initially a cattle virus, it is occurring also in small ruminants, in which it was neglected for a long time due to its low prevalence and insignificant clinical manifestation (Amer et al., 2019). In Australia, sheep were reported in 1978 to excrete CoV‐like particles that were visualized by electron microscopy examination during a diarrhoea episode (Tzipori, Smith, Makin, & McCaughan, 1978). The infection of small ruminants by the BCoV is rather to occur through close contact with cattle in mixed flocks than as a natural infection (Tråvén, Carlsson, Lundén, & Larsson, 1999). The most recent study in Ghana reported anti‐BCoV antibodies in 26 out of 102 (25.5%) and 29 out of 66 (43.9%) sheep and goats, respectively, associated to lambs’ newborn diarrhoea and respiratory infections (Burimuah et al., 2020) (Table 2).

### 3.2 Coronaviruses of Camelidae

#### 3.2.1 Dromedaries

Nowadays, it is well established that dromedaries are the natural host of the MERS‐CoV and a source for human and other domestic animals infections (Kandeil et al., 2019). Since the first case of human infected by the MERS‐CoV was identified in September 2012 in Saudi Arabia (World Health Organization, 2019), interest to dromedaries as sources of the virus increased and the isolated strains were shown to be genetically very similar to those isolated from humans (Omrani, Al‐Tawfiq, & Memish, 2015).

The close contacts between infected dromedaries and humans enhance the continuing zoonotic transmission and may explain why the MERS‐CoV continues to occur in humans (De Wit et al., 2016). Moreover, the MERS‐CoV was also detected in sheep, goats and donkeys reared close to camels (Kandeil et al., 2019).

Transmission from dromedaries to humans occurs by direct contact with mucous and nasal discharge or by consumption of meat or raw milk (Gossner et al., 2016; Mirkena et al., 2018). Indeed, MERS‐CoV RNA was detected using qRT‐PCR in whole and skimmed milk collected from milking animals that were kept with camels that have frequent contact with multiple origin camels participating at racing events (Reusken, Farag, et al., 2014).

The MERS‐CoV detection in dromedaries is difficult as the infection is asymptomatic. However, experimental MERS‐CoV infection of dromedaries caused mild to moderate rhinitis with nasal discharge, tracheitis and bronchitis in addition to the shedding of a large amount of virus from the respiratory tract (Haverkamp et al., 2018).

Several studies showed that the wild strains of the MERS‐CoV circulate in dromedaries in more than 24 countries across Africa and the Middle East (Figure 2), with high seroprevalences rates recorded from Egypt (71%) (Ali et al., 2017), Nigeria (94%) (Reusken, Messadi, et al., 2014) and Saudi Arabia 84% (Harrath & Abu Duhier, 2018). It was shown that the seroprevalence among dromedaries increases with age, reaching 80%–100% in adults (Harrath & Abu Duhier, 2018).

In experimentally challenged dromedaries, a modified orthopox‐based vaccine expressing the MERS‐CoV spike protein conferred mucosal immunity. This vaccine induced a significant reduction of virus shedding, and the conferred protection was associated with neutralizing antibodies production (Haagmans et al., 2016). As a strategy to prevent human contaminations, mass vaccination of camels is discussed (Dighe et al., 2019).

#### 3.2.2 Alpacas and llamas

Following a severe diarrhoea outbreak that induced high morbidity and mortality among neonatal alpacas (*Vicugna pacos*) in Silly (Southern Peru), 40% (20/50) of samples collected from dead alpacas were positive to CoVs using RT‐PCR (Rojas et al., 2016). In Oregon (USA), 39 out of 45 diarrheic crias (young llamas and alpacas) were positive to CoVs, based on morphological characterization of the virus after potassium phosphotungstate staining (Cebra et al., 2003). Coronavirus was also suspected to cause respiratory disease among alpacas in California, ranging from mild to fatal syndromes (Crossley et al., 2010). Reusken et al. (2016), found that 15 healthy alpacas in Al‐Shahaniya (Qatar) were seropositive to MERS‐CoV whereas both faecal and nasal swabs of the tested animals were negative by molecular assays. Phylogenetic analysis showed that the alpaca CoV genome displayed high identity (> 99.5%) with those of BCoV strains (Jin et al., 2007).

Today, interest to llamas increased as their antibodies could be engineered to block the attachment of the SARS‐CoV‐2 to the angiotensin‐converting enzyme (ACE2) receptor present on the human cell membranes of type 2 pneumocytes and intestinal epithelial cells (Dong et al., 2020).

### 3.3 Coronaviruses of Equidae

The equine coronavirus (ECoV) was isolated for the first time from a foal in North Carolina (USA) (Guy, Breslin, Breuhaus, Vivrette, & Smith, 2000), since then, multiple outbreaks were documented in different countries but epidemiological information on ECoV still scanty (Pusterla, Vin, Leutenegger, Mittel, & Divers, 2016). The ECoV was reported from Japan, Saudi Arabia, France, the United Kingdom and the United States (Table 2). Phylogenetic analyses showed that ECoV isolated from Japan and the United States, were genetically close (Nemoto et al., 2015).

Outbreaks were reported in almost all the US States (excepting Alaska and Hawaii). Between 2011 and 2017, 27,5% (130/472) positive qRT‐PCR were reported among horses’ samples pooled during 20 outbreaks in the United States (Pusterla et al., 2013). In Europe, the first detection of ECoV was made in France, during the winter season of 2011/2012 (Table 2) (Miszczak et al., 2014). Samples were taken from foal and adult horses suffering of mild respiratory and enteric conditions; eight out of 58 sampled French counties, comprised at least one positive sample (Miszczak et al., 2014).

The morbidity rates of ECoV infection ranged from 10% to 83% and the lethality rate is classified from rare to 27% (Fielding et al., 2015; Oue et al., 2011; Oue, Morita, Kondo, & Nemoto, 2013; Pusterla et al., 2013). Up to 83% of infected horses remain asymptomatic, while their faeces contain the ECoV (Pusterla et al., 2013).

The transmission route of the ECoV occurs through a faecal‐oral route (Pusterla et al., 2013) and the disease occurs mainly in winter (Nemoto et al., 2014). The ECoV could be detected from diarrheic horses 2 to 5 weeks after the beginning of the infection (Pusterla et al., 2016) but also from healthy horses (Hemida, Elmoslemany, et al., 2017). The peak of faecal shedding occurs 3 to 4 days after the onset of the disease (Bryan et al., 2019) and nasal secretions could be positive to the ECoV at this moment, but the epidemiological role of these secretions in transmitting the virus is not known (Nemoto et al., 2014). According to the observations of Pusterla, Vin, Leutenegger, Mittel, and Divers (2018), draft horses showed higher infection rate than other breeds, this could be explained by the stress induced by working conditions.

Some attempts of vaccination using the BCoV gave promising results but need further investigations (Nemoto et al., 2017).

### 3.4 Coronaviruses of Suidae

Six coronaviruses were isolated from pigs belonging to three genera: four to *Alphacoronavirus* (the porcine epidemic diarrhoea virus (PEDV), the transmissible gastroenteritis virus (TGEV), the porcine respiratory coronavirus (PRCV) and the severe acute diarrhoea syndrome virus (SADS**‐**CoV), one *Betacoronavirus* (the porcine hemagglutinating encephalomyelitis virus (PHEV)) and one *Deltacoronavirus* (the porcine deltacoronavirus (PDCoV))(Wang et al., 2019). Among these swine CoVs, the PHEV, the PEDV, the TGEV, the PRCV are known before 1984, whereas, the PDCoV and the SADS‐CoV are considered as emergent viruses (Table 1), beside a re‐emerging highly virulent strains of the PEDV that were described since 2010 (Sun et al., 2012).

#### 3.4.1 Transmissible gastroenteritis virus (TGEV)

The TGE is the sole mammalian disease caused by a CoV in the World Organisation of Animal Health (OIE) list (www.oie.int, Chapter 3.8.10.). TGE was reported since 1946 in the United States (Doyle & Hutchings, 1946). Then, it was reported in several regions including European countries, North, Central and South America, Southeast Asia: China, Japan, Korea, Nepal, Myanmar (Burma) and South and West Africa (Chen et al., 2019).

The virus could contaminate a non‐infected herd through the introduction of asymptomatically infected animal, then TGEV is transmitted by faecal–oral route (MacLachlan & Dubovi, 2017). Large amounts of TGEV are present in the faeces of infected pigs, lasting up to 18 months, beside virus milk shedding (Piñeyro et al., 2018). Domestic animals such as cats and dogs may play a role as host for the TGEV (Sestak & Saif, 2002).

As for most of mammalian CoVs, the TGE occurs during winter after an incubation period varying between 18 hr and 3 days (Pensaert, 1976). The course of the disease is characterized by vomiting and profuse diarrhoea and is marked by high morbidity and mortality rates mainly among piglets (Animal Health Australia, 2016). Although pigs of all ages are susceptible to the TGEV infection, animals older than 5 weeks display milder clinical symptoms than piglets (Piñeyro et al., 2018).

As the TGEV shares common epitopes for neutralizing antibodies with PRCV, a significant decrease of the TGE incidence occurred as a consequence of the cross protection conferred by the PRCV (Wesley & Woods, 1996).

Bivalent or trivalent live‐attenuated vaccines, combined with rotavirus, PEDV and/or *Escherichia coli* are available in Europe and North America. They are recommended for sows during gestation in order to provide lactogenic immunity to newborn piglets. Inactivated vaccines are mostly used in Asia (Gerdts & Zakhartchouk, 2017).

#### 3.4.2 Porcine hemagglutinating encephalomyelitis virus (PHEV)

The emergence of the PHE was traced back to 1957 in Canada, with the occurrence of high several episodes of vomiting, wasting and anorexia followed by neurological signs in pig nurseries (Roe & Alexander, 1958). In 1962, the virus was isolated from baby pigs suffering from encephalomyelitis (Greig et al., 1962), then several outbreaks with similar clinical signs were reported in Canada and Europe and the disease was named vomiting and wasting disease (VWD) (Cartwright et al., 1969). In 1971, the PHEV was classified as a CoV (Greig et al., 1971) and subsequently the disease was reported from numerous countries, like China, South Korea, Japan, Belgium, Canada, the United States and Argentina (Mora‐Díaz, Piñeyro, Houston, Zimmerman, & Giménez‐Lirola, 2019). Recently, the PHEV was detected and characterized for the first time in Czech Republic in 7.9% of pigs’ nasal swabs from different age categories (Moutelikova & Prodelalova, 2019). The PHEV circulates in swine populations in silent way infecting animals after replacement or weaning and the transmission occurs via nose‐to‐nose contact, by the inhalation of infected nasal secretions (Saif et al., 2019).

The PHEV infects all naive pigs at any age, but the clinical manifestations are most severe in piglets under 4 weeks of age, mainly born from naive dams, with mortality rate reaching 100% (Mora‐Díaz et al., 2019). Currently the PHEV infection is most likely to remain subclinical in affected herds, because the good protection conferred by the colostral immunity transferred to newborn piglets, which is considered as the best way to prevent from the infection (Rho et al., 2011).

#### 3.4.3 Porcine epidemic diarrhoea virus (PEDV)

The porcine epidemic diarrhoea (PED) was reported for the first time in England in 1971, but the viral aetiology was only proved in 1978 (Pensaert & de Bouck, 1978). The PEDV spread to other European countries (Belgium, France, Hungary and Czech Republic…) (Pensaert & Martelli, 2016) and to Asia where it became enzootic particularly in most prosper pork industry countries like China, South Korea and Philippines (Song & Park, 2012). The economic losses induced by the occurrence of the PED are dramatic since, high mortality rate is associated to the infection, mainly among < 10 days old piglets (Antas & Woźniakowski, 2019). Mortality reached 100% in newborn and suckling piglets in Japan during 1993 and 1994 (Sueyoshi et al., 1995) and in Thailand during 2007 and 2008 (Puranaveja et al., 2009). In the United States, the PEDV emerged in 2013, where almost 10% of the pig population died in less than 1 year after several outbreaks (Chen et al., 2014; Stevenson et al., 2013) and spread to Mexico and Canada in 2014 (Kochhar, 2014). Based on the study of Zhou et al. (2018), the PEDV was ranked as the most important causative agent of porcine diarrhoea, with an infection rate of 78.25%. The transmission of PEDV is faecal–oral and both contaminated vehicles and food was incriminated in PEDV spreading in the United States (Bowman, Krogwold, Price, Davis, & Moeller, 2015; Lowe et al., 2014; Pillatzki et al., 2015).

Due to the severity of the PED and its huge economic impact, several Asian countries are immunizing pigs with killed or live virus vaccines. Killed virus vaccine induced the higher antibody (IgA and IgG) levels than live virus vaccine particularly in sow's colostrum, sow's sera and piglets sera after suckling (Paudel et al., 2014). In Japan, live attenuated virus is used since 1997 (Usami, Yamaguchi, & Kumanomido, 1998) and oral vaccination started in South Korea and Philippines, since 2004 and 2011 respectively (Garcia et al., 2018; Park et al., 2018). As new virulent strains are circulating, trials for more effective vaccine are undergoing using swine‐poxvirus‐based vaccines designed to express the A epitope of the spike protein (Yuan, Lin, Li, He, & Fan, 2017).

#### 3.4.4 Porcine respiratory coronavirus (PRCV)

The porcine respiratory coronavirus (PRCV) is a mutant of the TGEV, due to a deletion in spike gene (Laude et al., 1993). It was identified first in Belgium in 1984 (Pensaert, Callebaut, & Vergote, 1986) and spread to almost all European countries including France (Madec et al., 2004), Denmark (Have, 1990) and the United Kingdom (Brown & Cartwright, 1986). The PRCV was soon reported from the United States (Halbur et al., 2003) and Japan (Usami et al., 1998).

Most of the PRCV infections are subclinical or inducing mild symptoms (Caswell & Williams, 2016), this could be due to the low proinflammatory cytokines synthesis activation (Van Reeth, Labarque, Nauwynck, & Pensaert, 1999). The PRCV is still detected upto the 21st day in the lungs of experimentally infected pigs and high titres reached 10^8.3^ median tissue culture infectious doses (TCDI50) per gram of lung tissue (Jung et al., 2007). Faecal shedding of PRCV was also reported from 37% (21/57) of sentinel pigs introduced in PRCV‐infected herds (Costantini et al., 2004).

Because of the insignificant clinical course of the PRCV infection, neither treatment nor vaccination, is applied. However, countries exporting live pigs need negative status to PRCV, which could be obtained only if piglets are pre‐weaned early at the 7th day, together with strict all‐in, all‐out managed barns, rigorous disinfection and cleaning of pig barns and regular seronegative tests in sows (Burlatschenko & Arsenault, 2015).

#### 3.4.5 Porcine deltacoronavirus (PDCoV)

The porcine deltacoronavirus (PDCoV) was first detected in asymptomatic pigs in 2009 in Hong Kong (China) during a molecular survey (Woo et al., 2012). However, another retrospective molecular study showed that the virus might circulate since 2004 in China (Dong et al., 2015). It was only in 2014 that the clinical signification associated to the PDCoV infection was addressed after multiple outbreaks occurred in Ohio State (USA) (Marthaler, Jiang, Collins, & Rossow, 2014; Wang, Byrum, & Zhang, 2014a). Then, the PDCoV spread to other American States (Iowa, Illinois, Minnesota and Nebraska), Canada (Wang, Byrum, & Zhang, 2014b), South Korea (Lee & Lee, 2014), mainland China (Dong et al., 2015), Thailand, Vietnam and Lao Popular Democratic Republic (Saeng‐Chuto et al., 2017).

According to Sun et al. (2020) the PDCoV infection became enzootic in certain region of China. Molecular prevalences ranged from 10.06% (17/169) to 16.9% (29/172), in Hong Kong and Taiwan respectively (Woo et al., 2012; Hsu et al., 2018). The co‐infection PDCoV‐PEDV is frequently reported in pigs’ farms where gastroenteric problems occur (Jang et al., 2017; Lee et al., 2016; Zhang, Liu, et al., 2019). According to Zhang, Liu, et al. (2019) suckling pigs were most infected than finishing pigs and the PDCoV was recovered from milk samples at the rate of 7.92% (8/101).

Epidemiological patterns of the PDCoV infection are similar to those of PEDV, but the reported prevalences remain lower in diarrheic pigs compared with those reported for PEDV and TGEV infections (Zhang, 2016). Despite mortality rates are lower than that usually observed for PEDV infection, the emergence of the PDCoV disease in Thailand in 2014, caused heavy economic loses to pork farms with a mortality rate reaching 64.27% (2,892/4,500) in piglets (Janetanakit et al., 2016).

Mixing different animal species in swine farms may play an important role in the epidemiology of the PDCoV infection, as chicks and poultry are susceptible to the PDCoV (Liang et al., 2019), thus it is not excluded that they may act as intermediate hosts for the virus. Moreover, in orally PDCoV‐inoculated calves, a persisting faecal viral RNA shedding associated to PDCoV‐specific serum IgG antibody responses were observed (Jung et al., 2017). There is no commercial available vaccine for the prevention from the PDCoV infection (Zhang, Liu, et al., 2019). Active surveillance programme effectively led to the decrease of the PDCoV herd‐level prevalence below 0.5% in two years in Canada (Ajayi et al., 2018).

#### 3.4.6 Swine acute diarrhoea syndrome virus

The swine acute diarrhoea syndrome virus (SADS**‐**CoV) has emerged in pigs in China since August 2016 (Zhou, Sun, et al., 2019). This virus, also qualified as Porcine entreric alphacoronavirus (PEAV) (Xu et al., 2019) or Swine enteric coronavirus (SeCoV) (Pan et al., 2017) was associated with the occurrence of severe diarrhoea of suckling piglets in Guangdong province in 2017 (Gong et al., 2017; Pan et al., 2017) and 2019 (Zhou, Li, et al., 2019), and in Fujian province in 2018 (Li et al., 2018). The economic loss induced by the SADS‐CoV was dramatically high, as 24,693 piglets in four farms, died from the infection in Guangdong province, where the first human SARS epidemic started in 2003 (Zhou et al., 2018). This virus is antigenically distinct from the PEDV, the TGEV and the PDCoV (Yang, Yu, & Huang, 2020). Nevertheless, the complete genome sequencing of the N and the S genes showed a high nucleotides homology (96%–98%) with those of four bat‐CoV HKU2 (Gong et al., 2017). During the first outbreak in China, the SADS‐CoV infection was controlled by immunizing pregnant sows using inactivated filtrated virus obtained from infected piglets intestines (Zhou, Sun, et al., 2019b). As there is an urgent need to develop efficient vaccines to control the SADS‐CoV in pigs, trials on attenuation of a virulent strain via cell culture passage are now on‐going (Sun et al., 2019).

#### 3.4.7 Recombinant emerging pigs’ coronavirus

decaA new chimeric virus containing the S protein of the PEDV on a TGEV backbone was discovered in Italy in 2016 and might circulate there from mid‐2009 to 2012 according to Boniotti et al. (2016). This recombinant virus was also detected in Germany, Central Eastern Europe and Slovakia precluding for an old circulation in Europe (Akimkin et al., 2016; Belsham et al., 2016; Mandelik et al., 2018).

### 3.5 Coronaviruses of Leporidae

In 2012, a novel Beta‐CoV was isolated from domestic rabbit and characterized as rabbit coronavirus HKU14 (RbCoV HKU14), causing no clinical manifestation. The virus was detected in 8.1% (11/136) rabbit faecal samples using RT‐PCR and phylogenetic analysis showed that the novel RbCoV HKU14 is most closely related to betacoronavirus 1 species (BCoV, ECoV, CCoV, HCoV‐OC43) (Lau et al., 2012). The authors hypothesized that the virus may have emerged as a result of interspecies transmission between different animal species in Chinese markets.

### 3.6 Cetacean coronaviruses

Three species of sea mammals were shown to be susceptible to CoVs: harbor seals, white beluga whale and bottlenose dolphins.

In 1987, three captive seals (*Phoca vitulina*) at the Miami Seaquarium expressed acute enteritis associated with dehydration and leucocytosis. The pathological examination showed bronchoalveolar oedema and haemorrhage. Fluorescent antibody staining yielded positive results to TGEV, FIPV and CCoV antisera, which precluded to an Alpha‐CoV, but the virus was not yet assigned to the genus (Bossart & Schwartz, 1990).

Ten years later, a captive beluga whale (*Delphinapterus leucas*), in the United States suffering from a generalized pulmonary disease, died from acute liver failure caused by a CoV (Mihindukulasuriya, Wu, St. Leger, Nordhausen, & Wang, 2008). Phylogenetic analyses revealed that the causative agent was closely related to the infectious bronchitis virus (IBV) of chickens, and the pathogen, named BWCoV SW1 was assigned to the Gamma‐CoV genus (de Groot et al., 2012).

In bottlenose dolphins (*Tursiops aduncus*), the first report of a CoV, was made by Woo et al. (2014) from an Ocean Park in Honk Kong and the virus was qualified as BdCoV HKU22. The virus was recovered from 3 out of 48 faecal samples collected on healthy bottlenose dolphins, whereas the tested samples from California sea lions (*Zalophus californianus*) and harbor seals kept at the same park were negative. Despite that the comparative genome analysis showed similar characteristics between the BdCoV HKU22 and the BWCoV SW1, difference was detected at the protein encoded by the S gene, as only 74.7% amino acid identities was found (Woo et al., 2014). During April 2019, four bottlenose dolphins kept by the US Navy Marine, displayed diarrhoea and anorexia. Phylogenetic analyses of the complete genome, showed that the four American BdCoVs were clustered with the three Hong Kong BdCoV HKU22 but distant from the avian CoVs (Wang et al., 2020).

### 3.7 Avian coronaviruses

Avian CoVs are the main representative of the Gamma‐CoV genus, including the IBV in chickens as the most studied CoV, and unique among all other animal CoVs. Similar CoVs to the IBV were detected and isolated from domestic galliformes: turkey (TCoV), guinea fowl (GfCoV) and pheasants (PhCoV) (Cavanagh, 2005), but also from non‐galliformes namely: Columbiformes, Psittaciformes and Anseriformes (Domanska‐Blicharz et al., 2014; Jonassen et al., 2005). Seven other avian CoVs are included in the Delta‐CoV genus (Woo et al., 2012). Only the IBV will be treated in this section.

The first isolation of the IBV was made in 1930s in the United States (Massachusetts) and it was thought that only one serotype exists, until 1956, when Jungherr et al. (1957) discovered that based on neutralization tests, there are different serotypes of the IBV. The IBV disease is the second one caused by a CoV to be listed among the notifiable diseases in the OIE (www.oie.int, Chapter 3.3.2.).

In 2016, Valastro et al. (2016) suggested a method to harmonize the nomenclature of the IBV using a phylogeny‐based classification and complete genome sequencing of the S1 gene. Today, the IBV is divided to seven genotypes including 35 distinct viral lineages based on the complete S1 gene sequencing (Ma et al., 2019; Xu et al., 2019).

The geographic distribution of the IBV is worldwide (Figure 2) and some genotypes are present more in certain regions than in others. Indeed, two different lineages GI‐21 and GII‐1 were considered as specific to Europe (Fan et al., 2019), while GI‐9, GI‐27 and GIV‐1 were considered specific to North America (Lin & Chen, 2017) and the GI‐11 is an exclusively South American lineage, very prevalent in Brazil and Uruguay (Marandino et al., 2019).

The IBV is a highly contagious virus of the upper respiratory tract of chickens, leading to 100% morbidity and a mortality rate ranging from 0% to 82% depending upon several factors such as the age of the birds, their immune status, and the involvement of secondary pathogens (Ramakrishnan & Kappala, 2019). The IBV infects chickens of all ages, although young are most susceptible, and may die directly from the IBV infection or from mixed infections mainly caused by *Escherichia coli*, leading to heavy losses to the breeding industry (Jackwood, 2012). In Brazil, which is considered as the biggest broiler meat exporter (Nääs, Mollo Neto, Canuto, Waker, Oliveira, & Vendrametto, 2015), the total loss (in US$) induced by the IBV infection were estimated per 1,000 birds to 4,210.8 and to 266.3, in breeders and broiler respectively (Colvero et al., 2015).

To control the IBV infection in avian farms, although insufficient, high level of biosecurity measures is required such as removing faeces from the premises, rigorous cleaning and disinfection and single‐age housing (de Wit & Cook, 2019). However, because of the high resistance of the IBV in the environment, vaccination is a helpful method. Nevertheless, because of the high recombination rate among the IBV strains, new lineages are constantly discovered, which makes control through vaccination very challenging. Moreover, there is poor cross reactions between the heterologous strains (de Wit et al., 2016). Attenuated live vaccines and killed vaccines, are used, in broilers and pullets and in layers and breeders, respectively, with few different types of IBV vaccines available for use (Jackwood, 2012). The application technic of the vaccine, the combination with other vaccines and the use of heterologous strain of IBV, are among the negative factors that might influence the vaccination success (de Wit & Cook, 2019).

## 4 PUBLIC HEALTH IMPLICATIONS IN RELATION WITH ANIMAL CORONAVIRUSES

Except the SARS‐CoV, the MERS‐CoV and the most recent SARS‐CoV‐2, the other documented human CoVs are specific to human beings (Corman et al., 2018). It is well established that domestic or wild animals were involved in the three epidemics either as reservoir or as intermediate hosts.

### 4.1 The SARS‐CoV epidemic

The SARS‐CoV epidemic started in China in April 2003, caused the infection of 8,422 persons and killed 916 worldwide (Chan‐Yeung & Xu, 2003). Several wild animals, including palm civets (*Paguma larvata*), raccoon dogs (*Nyctereutes procyonoides*) and horseshoe bats (*Rhinolophus hipposideros*) were involved in the SARS‐CoV epidemic during 2003 and were tested positive using virological and or serological tests (Guan et al., 2003). While phylogenetic analyses showed that bats are reservoir for the SARS‐CoV and allowing genetic recombination, civets seem to be an intermediate host, as they were tested negative in their wild free lands (Su et al., 2016).

### 4.2 The MERS‐CoV epidemic

The MERS‐CoV epidemic has emerged from Saudi Arabia in June 2012 and was distributed to 27 countries in the four continents: Asia, Europe, Africa and America. From 2012 to July 2019 the MERS‐CoV infected 2,449 persons and caused the death of 845 (World Health Organization, 2019). Testing of archived dromedary sera precluded for the circulation of the MERS‐CoV since at least three decades in Saudi Arabia (Alagaili et al., 2014; Hemida et al., 2014).

Despite no contact history of infected persons with animals was reported, camels in several East and African countries expressed neutralizing antibodies against MERS‐CoV and the virus was isolated from their nasal mucous (De Wit et al., 2016). The hypothesis of bat origin was advanced based on CD26 receptor used by both MERS‐CoV and bat‐CoV HKU4 to enhance cell entry, but to date neither the MERS‐CoV has been isolated from bats, nor its RNA was detected (Omrani et al., 2015). Sheep, goats, cattle, donkeys that were in close contact with confirmed positive dromedaries, showed high titres in neutralizing antibody test in both Egypt and Senegal and were further confirmed carrying MERS‐CoV RNA as detected by RT‐PCR (Reusken et al., 2013). Recent published study on modelling the MERS‐CoV sequences data, showed that the MERS‐CoV evolve exclusively in camels while humans act as terminal host (Dudas et al., 2018). The epidemiology of MERS remains to date not well understood, as heavily exposed persons to infected camels lead only to seropositivity and any of persons confirmed with the MERS‐CoV reported previous exposure to infected animals (Hemida, Elmoslemany, et al., 2017). The main factor that could play a role in the transmission of MERS‐CoV from infected dromedaries to humans, is the consumption of raw camel milk (Reusken, Farag, et al., 2014). Neither the consumption of meat and organs nor the consumption of urine for medicinal use might cause the infection of humans (Adney et al., 2014) and further investigations on experimentally inoculated animals are needed to confirm such definite conclusions.

### 4.3 The SARS‐CoV‐2 epidemic

For the current COVID‐19 epidemic, the bat involvement as advanced by (Zhou et al., 2020) is not excluded, as the SARS‐CoV‐2 displayed 96% identity to the whole genome of bat‐ CoV. Moreover the SARS‐CoV‐2 was closely related to five wild animal CoVs, including civets and pangolin (Li et al., 2018). Further studies are needed to trace back the whole transmission chain and to understand the real involvement of intermediate animals in this unpreceded epidemic, as contact between bats and human is less likely to occur in nature.

In the other side, the SARS‐CoV‐2 infection was notified to the World Organisation of Animal Health (OIE) from domestic and captive wild animals. Nasal, oral and faecal swabs of two dogs and one cat belonging to COVID‐19‐positive owners were tested positive to the SARS‐CoV‐2 using qRT‐PCR and virus isolation in several districts in Hong Kong. The dogs and cat did not show any clinical signs of infection and were returned to their owners when they tested negative (World Organisation of Animal Health, 2020). In Bronx (New York, USA), five tigers (*Panthera tigris*) and three lions (*Panthera leo*) maintained in a zoo showed symptoms of COVID‐19, including dry cough and anorexia. One animal tested positive to the SARS‐CoV‐2 and it was hypothesized that an asymptomatic employee might transmit the virus to these wild animals (World Organisation of Animal Health, 2020).

In addition, the occurrence of respiratory disease and increasing mortality caused by the SARS‐CoV‐2 infection in mink farms were registered in the Netherlands since April 2020 (Oreshkova et al., 2020). Further outbreaks appeared in four mink farms located in North Jutland in Denmark (https://www.foedevarestyrelsen.dk/) and in Spain where the government ordered the culling of almost 100,000 animals to avoid the spread of the virus (https://www.bbc.com/news/world‐europe‐53439263). On 17th August 2020, the United States Department of Agriculture announced the first case of the SARS‐CoV‐2 in mink farms in Utah State, where animals most likely had exposure to a confirmed human with COVID‐19 (United States Department of Agriculture, 2020).

Experimental studies showed that ferrets and cats are highly susceptible to the SARS‐CoV‐2. After an intra‐nasal inoculation of 10^5^ plaque‐forming units (PFU) to multiple animal species including dogs, cats and ferrets, the infectious virus was detected in nasal turbinate, soft palate and tonsils of all inoculated ferrets. The SARS‐CoV‐2 RNA was also detected in rectal swabs and efficacious droplet transmission was confirmed among exposed cats (Shi et al., 2020).

The phylogenetic studies associated to amino acid sequence comparison of the ACE2 receptor has predicted cow, buffalo, goat, sheep and swine, as well as other wild species as the potential intermediate hosts for the SARS‐CoV‐2 (Qiu et al., 2020). These findings highlighted the threat from the interspecies transmission of the SARS‐CoV‐2 between multiple animal species, and from animals to human and *vice versa*.

### 4.4 Factors to take in consideration to prevent further epidemics

Multiple factors are playing an important role in increasing the exposure of humans to CoVs of infected animals.

#### 4.4.1 Agriculture intensification and environmental changes

Intensification of livestock production implies increasing animal density in farms and increasing movement of human and vehicles on and off farms, which promotes pathogen transmission and spreading (Cutler, Fooks, & van der Poel, 2010). The intensively development of swine industry the last decade is considered of high risk for the trigger of severe pandemic viruses for humans (Borkenhagen, Salman, Ma, & Gray, 2019). This was shown particularly with swine influenza viruses, and Nipah virus. Indeed, swine exposed workers displayed significantly higher titers (in hemagglutination inhibition assay) against swine influenza subtypes H1N1 and H1N2 than non‐exposed workers (Odds Ratio = 54.9 and 95% confidence interval: [13.0–232.6] (Gray et al., 2007). In late 1998 and 1999, several fatal encephalitis cases (*n* = 105) caused by a Paramyxovirus were recorded in Malaysia (Chua, 2000) and quickly spread to Singapore (Paton et al., 1999). Respiratory disease and encephalitis were recorded few weeks ago in pigs in the same district and the isolated virus, named Nipah virus, was shown to be transmitted from bats to pigs, then to humans (Chua et al., 2002).

The consequences of anthropogenic activities leading to deforestation, habitat fragmentation and replacement of natural vegetation by crops definitely modify wildlife biology. These anthropogenic activities foster wildlife migration and create new environments that increase host, vector and pathogen contacts (Jones et al., 2013). Indeed, the deforestation was incriminated in the occurrence of the Nipah virus outbreak by pushing fruitbats (the natural reservoir of the Nipah virus) to pig farms high‐density areas and fostered the contact between both species (Chua et al., 2002).

#### 4.4.2 Trade of domestic and wild animals

It is well established that animals and animal product trade foster pathogens spreading (Travis, Watson, & Tauer, 2011). A study on livestock trade network assessment in Europe, showed that pigs (for fattening and slaughter) and cattle (for fattening) were ranked second as the most heavily moved animal species within Europe in 2011, which foster the risk for exotic diseases introduction (Hardstaff, Häsler, & Rushton, 2015).

Thousands of wild animals are traded in Asian markets every day, such as raccoon dogs, pangolin, masked palm civets, ferret badgers and hedgehogs (www.animalasia.org). As several domestic and wild animals were shown to harbour CoVs, handling, touching or eating these animals, could increase the risk for humans to be in contact with CoVs.

#### 4.4.3 Changing in the distribution of invasive animal species

Rodents have a worldwide distribution and were shown to harbour more than 60 known pathogens infecting both animals and humans (Meerburg, Singleton, & Kijlstra, 2009), including CoVs (Wang et al., 2015). Bats also are considered to be the most abundant and diverse vertebrate after rodents (Rodhain, 2015), with more than 1,300 known species and worldwide geographic distribution (Teeling, Jones, & Rossiter, 2016). Bats are considered as an important reservoir of highly lethal zoonotic viruses and were shown to harbour more CoVs than any other animal species (Hu et al., 2015). Indeed, beside the SARS‐CoV, the SARS‐CoV‐2 and the MERS‐CoV, two porcine CoVs, have a bat‐CoV origin: the SADS‐CoV and the PEDV (Banerjee, Kulcsar, Misra, Frieman, & Mossman, 2019). The geographical range of some bat species was estimated to expand by near to 400% in the last four decades as a response to climate change (Ancillotto, Santini, Ranc, Maiorano, & Russo, 2016). One could imagine, that this expansion would be favourable to a higher contact likelihood with wild and domestic animals.

#### 4.4.4 Herd management practices

Mixing different animal species would increase CoVs host jumping probability. The BCoV contaminates sheep and goats, when they share the same barns or graze with infected cattle (Tråvén et al., 1999). The MERS‐CoV was also detected from sheep, goats and donkeys kept in close contact with infected dromedaries (Reusken et al., 2013).

## 5 CONCLUDING REMARKS

The ability of CoVs to mutate and to adapt to a new environment makes them a continuous threat to human lives and to domestic animals, mainly of farm industries. Pig CoVs are well described and documented in part because pork meat is the most widely consumed in several countries around the world. Nevertheless, data on several animal CoVs are scarce from multiple countries, mainly from Africa. There is an urgent need to characterize the extent of CoVs infections among domesticated animals in the world using serological investigations for screening, virological studies as confirmatory and phylogenetic studies as comprehensive of the mutation and recombination phenomena. Continuous surveillance programme for CoVs genetic evolution should be implemented and would serve for at risk areas prediction. To date, only one mammal animal CoV is registered in the OIE list, namely the TGEV and one avian, the IBV. More attention should be given to the other animal CoVs and a specific surveillance system must be implemented in all the OIE member countries to monitor the introduction and the emergence of novel CoV strains in new areas. Strict regulation is to be implemented for farm animal trade. Thus, it is urgent to assess if meat, milk, semen and other animal by‐products are at risk for humans, either through manipulation, process or consumption.

The accumulated knowledge needs to be compiled, broaden and extensive to predict and prevent further fatal pandemic like the COVID‐19.

## 6 ETHICS STATEMENT

No ethical approval was required as this is a review article with no original research data.

## CONFLICT OF INTEREST

The authors declare that they have no conflict of interest.

## AUTHOR CONTRIBUTION

**Mediha Khamassi Khbou:** Conceptualization; Methodology; Supervision; Writing‐original draft; Writing‐review & editing. **Monia Daaloul Jedidi:** Writing‐original draft; Writing‐review & editing. **Faten Bouaicha:** Writing‐original draft; Writing‐review & editing; Construction of the phylogenetic tree.
